# Heat Shock Affects Mitotic Segregation of Human Chromosomes Bound to Stress-Induced Satellite III RNAs

**DOI:** 10.3390/ijms21082812

**Published:** 2020-04-17

**Authors:** Manuela Giordano, Lucia Infantino, Marco Biggiogera, Alessandra Montecucco, Giuseppe Biamonti

**Affiliations:** 1 Istituto di Genetica Molecolare, Consiglio Nazionale delle Ricerche via Abbiategrasso 207, 27100 Pavia, Italy; ma.giordano@mail.scuole.vda.it (M.G.); lucia.infantino@igm.cnr.it (L.I.); alessandra.montecucco@igm.cnr.it (A.M.); 2 Dipartimento di Biologia e Biotecnologie, Università di Pavia, 27100 Pavia, Italy; marco.biggiogera@unipv.it

**Keywords:** heat shock, non-coding RNAs, nuclear stress bodies, chromosome segregation

## Abstract

Heat shock activates the transcription of arrays of Satellite III (SatIII) DNA repeats in the pericentromeric heterochromatic domains of specific human chromosomes, the longest of which is on chromosome 9. Long non-coding SatIII RNAs remain associated with transcription sites where they form nuclear stress bodies or nSBs. The biology of SatIII RNAs is still poorly understood. Here, we show that SatIII RNAs and nSBs are detectable up to four days after thermal stress and are linked to defects in chromosome behavior during mitosis. Heat shock perturbs the execution of mitosis. Cells reaching mitosis during the first 3 h of recovery accumulate in pro-metaphase. During the ensuing 48 h, this block is no longer detectable; however, a significant fraction of mitoses shows chromosome segregation defects. Notably, most of lagging chromosomes and chromosomal bridges are bound to nSBs and contain arrays of SatIII DNA. Disappearance of mitotic defects at the end of day 2 coincides with the processing of long non-coding SatIII RNAs into a ladder of small RNAs associated with chromatin and ranging in size from 25 to 75 nt. The production of these molecules does not rely on DICER and Argonaute 2 components of the RNA interference apparatus. Thus, massive transcription of SatIII DNA may contribute to chromosomal instability.

## 1. Introduction

Centromeres are structural chromatin domains responsible for the faithful segregation of chromosomes during cell division. They consist of two distinct regions: the centromere core flanked on both sides by pericentromeric domains. Intriguingly, both the centromeric and pericentromeric sequences are poorly conserved in evolution except for their enrichment in highly repetitive, mainly satellite, DNA elements [1]. Centromeres have long been considered as constitutively heterochromatic and transcriptionally silent portions of the genome. This view has been disproved by the identification of both pericentromeric and centromeric transcripts in several organisms [2,3]. Although the function of these molecules is not completely understood, they appear to have a major role in establishing the epigenetic signature of pericentromeric and centromeric domains [4,5,6,7,8,9]. Breakthrough studies in the yeast *S. pombe* have proven that this function relies on the processing of centromeric transcripts into short RNA molecules by the RNAi machinery [4,10]. The strongest evidence of the involvement of the RNAi machinery in the processing of pericentromeric RNAs and heterochromatin assembly in vertebrates has been provided by Fukagawa et al. [11]. They found that loss of DICER in a chicken-human hybrid cell line carrying human chromosome 21 produces the accumulation of long pericentric Satellite III transcripts, and causes mitotic defects due to precocious sister chromatid separation. However, the validity of this mechanism in mammalian cells is still weak since short pericentromeric RNAs in vertebrate cells are difficult to detect. On the other hand, the role of pericentromeric transcripts in the assembly, maintenance and function of heterochromatin in mammals is substantial [12]. It is noteworthy that in primary mouse embryonic fibroblasts, transcription of pericentromeric heterochromatin is controlled during the cell cycle [13] with the production of long, heterogeneous transcripts in G1 and a pool of shorter transcripts (∼200 nt) at mitotic onset.

Not surprisingly, the transcriptional and epigenetic status of pericentromeric satellite sequences is altered in many cancers and genetic disorders [14,15,16,17,18,19,20]. A large increase in major, pericentromeric satellite transcripts in mouse cells [14] is accompanied by the loss of the heterochromatic state and by genomic instability, such as chromosome breaks and bridges. Interestingly, in both mouse and human the expression of pericentromeric satellite sequences is greatly increased in tumor compared to normal tissue [17]. Because of the poor conservation of pericentromeric sequences across species, this raises the question as to whether pericentromeric RNAs themselves or the transcription process are relevant to genome stability [21].

A few years ago, we and the group of Claire Vourc’h independently showed that heat shock induces the massive transcription of long arrays of Satellite III (SatIII) sequences in pericentromeric domains of several human chromosomes, the largest of which being on chromosome 9 (HSA9) [2,22,23,24]. A very low basal level of SatIII RNAs is detectable using quantitative PCR, but not using Northern blotting, even in unstressed cells [25]. Both strands of the repeats are transcribed; however, a strong bias in favor of G-rich SatIII transcripts is detectable both in unstressed and heat-shocked cells. Thus, the term SatIII RNAs normally designates G-rich RNAs. Production of SatIII RNAs can be elicited by a number of stressing conditions, such as heat shock, heavy metals, UV light and hyperosmotic or oxidative stress. The level of induction depends on the nature, intensity and length of stressing treatments [25], which suggests that transcriptional activation of SatIII sequences could be part of a general response to stress.

SatIII RNAs remain associated with sites of transcription and act as Architectural (arc) RNAs in the assembly of specific membrane-less, phase-separated compartments [26] called nuclear stress bodies or nSBs [27]. Electron microscope studies have shown that nSBs correspond to clusters of perichromatin granules (PG), i.e., highly packed forms of ribonucleoprotein complexes [28]. In addition to SatIII RNAs and DNA, nSBs contain acetylated histones and transcription (HSF1, RNAPII, CREB) and pre-mRNA processing factors [27,29]. The observation that SatIII RNAs mediate the recruitment to nSBs of a number of RNA binding proteins such as SRSF1 (SF2/ASF), SRSF7 (9G8), and SRSF9 (SRp30) and hnRNP Hap and M [29] led researchers to hypothesize a role of these RNAs in controlling pre-mRNA splicing [29,30]. This hypothesis has been recently verified by the Hirose group [31]. Using mass spectrometry analysis of ribonucleoprotein complexes assembled in vivo on SatIII RNAs, they have identified 141 proteins as putative nSB components in HeLa cells. This set includes CLK1, a kinase that phosphorylates SR splicing factors (SRSF1, SRSF7 and SRSF9) which are de-phosphorylated upon heat shock. SatIII RNA would act as a platform to sequester de-phosphorylated SR factors and to accelerate their re-phosphorylation by CLK1 and the ensuing release from nSBs. The consequence of this event is the alteration in the splicing profile, mainly an increase in intron retention events, of a specific set of gene transcripts during the first four hours of recovery from mild heat shock. Interestingly enough, phosphorylation of SRSF9 appears to have a crucial role in this phenomenon [31]. In addition to the impact on gene expression at the level of splicing regulation, SatIII RNAs can suppress transcription of a subset of genes by sequestering the histone acetyltransferase CBP to nSBs [32]. Interestingly, the protein composition of nSBs appears to be highly dynamic, and factors involved in transcription are no longer detectable after 3 h of recovery from moderate stress, whereas splicing factors persist up to 12 h [29].

In this manuscript, we investigate the complex life of SatIII RNAs and nSBs. SatIII RNAs are detectable for a long time period in human heat-shocked HeLa cells and are eventually processed into a new class of small RNAs that persist in close association with chromatin. Moreover, we have found that heat shock alters mitotic progression and induces lagging chromosomes (LCs) and chromosome bridges (CBs) in anaphase/telophase. In particular, upon stressing conditions, SatIII RNAs and nSBs often mark chromosomes undergoing segregation defects in mitosis. Chromosomal instability (CIN) has been characterized as one of the most relevant causes of aneuploidy in human cells [33] and correlates with a high rate of segregation defects during mitotic cell division [34]. Our finding that heat shock and massive transcription of pericentromeric SatIII affect the mitotic behavior of human chromosomes opens new perspectives on the analysis of chromosome instability.

## 2. Results

### 2.1. Satellite III RNAs Mark a Long Period of Recovery from Stress

**Figure 1:**
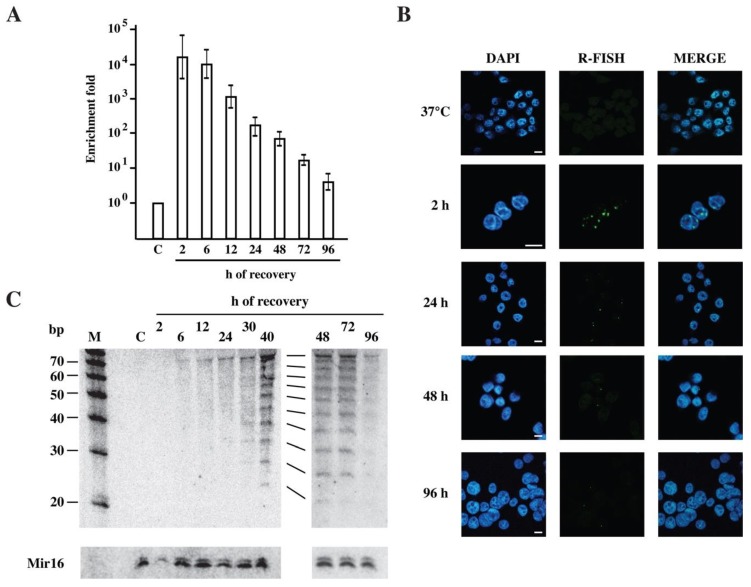
SatIII RNAs mark a long period of recovery from stress. (**A**) Total RNAs were extracted from unstressed HeLa cells (C) and from cells heat shocked for 1 h at 42 °C and then allowed to recover at 37 °C for the indicated time periods. The expression level of SatIII RNAs was determined using real time PCR, as described [25], and expressed as a function of the basal level observed in control cells. The histogram represents the average of three independent experiments ± standard deviation. (**B**) The same cell populations were analyzed using in situ RNA FISH with a biotinylated oligo against SatIII RNAs, as previously described [23]. Cell nuclei were stained using DAPI. (**C**) Short RNAs were prepared from HeLa cells treated as in panel A, separated on 5% polyacrylamide gels and analyzed using Northern blotting with a locked nucleic acid (LNA) oligo specific for SatIII repeats. M: molecular size markers. The same filter was hybridized to a probe specific for Mir16 as a loading control.Bars: 10 µm.

Heat shock induces the activation of arrays of repetitive Satellite III (SatIII) DNA and the massive production of long non-coding SatIII RNAs that remain associated with sites of transcription giving rise to nuclear stress bodies (nSBs) [23,29]. Although SatIII RNA sequences are detectable using quantitative RT-PCR, even in unstressed cells, their abundance drastically increases after heat shock and peaks between 2–6 h of recovery when a 10^4^–10^5^-fold induction is consistently observed [25]. As shown in Figure 1A, and in agreement with our previous analysis [25], a conspicuous (about 90%) reduction in the SatIII RNA level occurs during the first 24 h of recovery. However, the remaining 10% is relatively stable, and a fraction of these molecules is detectable even at 96 h of recovery.

A similar profile can be observed when SatIII RNAs are visualized using in situ hybridization with a biotinylated oligonucleotide probe [23] that marks nuclear stress bodies (nSBs) (Figure 1B). Altogether these analyses suggest the existence of three phases in the life cycle of SatIII RNAs. During phase I (from 0 to 6 h of recovery) SatIII DNA arrays are heavily transcribed giving rise to numerous and large nSBs, detectable in most of the cell nuclei. In phase II (from 6 to 24 h) the level of the SatIII RNAs drops and only 1–2 small nSBs can be visualized in the majority of the cells. Finally, during phase III a progressive reduction in the SatIII RNA level is observed, which is accompanied by a decreased fraction of cells with nSBs.

### 2.2. Long Satellite III RNAs Are Processed into Short Molecules During Phase III of the Heat Shock Response

In the fission yeast *Schizosaccharomices pombe* non-coding RNAs deriving from transcription of pericentromeric repeats are processed using DICER ribonuclease, into short RNAs that direct the transcriptional silencing of complementary repetitive DNA elements [35]. It has been proposed that a similar mechanism operates in higher eukaryotes to control the formation of large blocks of heterochromatin [11]. However, small pericentromeric RNAs in human and mammalian cells are difficult to detect and study.

**Figure 2:**
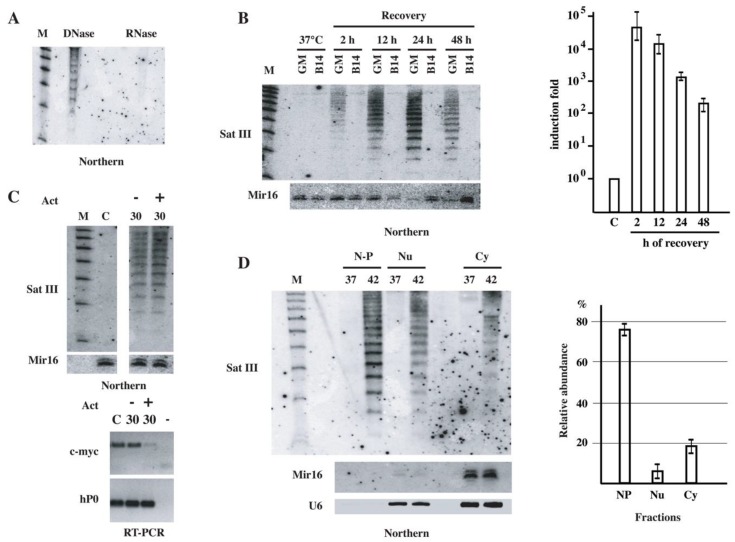
Characterization of short SatIII RNAs. (**A**) Short RNAs were prepared from heat-shocked HeLa cells allowed to recover at 37 °C for 42 h. RNAs were digested with DNase I or with RNase A and then analyzed using Northern blotting with the LNA oligo specific for SatIII repeats. (**B**) Short RNAs were prepared from heat-shocked hamster B14-150 cells and from the hamster>human somatic cell hybrid GM-10611A containing human chromosome 9. RNAs were then analyzed using Northern blotting as in panel A. Hybridization to Mir16 was used as a loading control. The histogram shows the quantitation of long SatIII RNAs in unstressed (C) and in heat-shocked GM10611A cells allowed to recover at 37 °C for the indicated time periods. Data are represented as mean fold increases ± standard deviation of three independent experiments. (**C**) Short RNAs were prepared from unstressed (C) and from heat-shocked (1 h at 42 °C) HeLa cells allowed to recover for 30 h at 37 °C in the presence or in the absence of Actinomycin D (5 μg/mL) during the last 6 h of recovery. The same membrane was hybridized with the LNA probe against SatIII RNAs and with a probe against Mir16 (loading control). M: molecular size marker. Total RNAs prepared from the same cells were analyzed using RT-PCR to assess the level of c-myc RNAs to control the efficacy of Act D treatment. RT-PCR analysis of hP0 RNA was used as a loading control. (**D**) Unstressed (37) and heat-shocked HeLa cells were collected after 40 h of recovery at 37 °C and fractionated in nuclear pellet (N-P), nucleoplasm (Nu) and cytoplasm (Cy) fractions. Total and short RNAs were prepared from each fraction. Short RNAs were analyzed using Northern blotting with an LNA probe against SatIII repeats. As a control of loading and fractionation quality, the same filter was hybridized to a probe against Mir16 (mainly cytoplasmic) and U6 RNAs. Total RNAs from nuclear pellet, nucleoplasm and cytoplasm fractions were prepared from heat-shocked HeLa cells allowed to recover for 40 h at 37 °C. RNAs were analyzed using quantitative RT-PCR. The histogram shows the relative abundance of SatIII RNAs in the three factions as determined in three independent experiments. Data are mean ± standard deviation.

We decided to investigate whether stress-induced SatIII RNAs could be eventually processed into short molecules. Therefore, small RNAs from unstressed, from heat-shocked (1 h at 42 °C) HeLa cells and from cells at different recovery times were analyzed using Northern blotting with a 20 nt locked nucleic acid (LNA) oligo, complementary to the (GGAAT)4 consensus sequence of SatIII transcript (SatIII-oligo). As shown in Figure 1C, a ladder of hybridization signals with discrete bands from 25 to about 75 nt is detectable in small RNA preparations from cells in phase III. These molecules appear at around 30 h of recovery, peak at 40–48 h and almost disappear at day 4. The regular 5 nt increment between the bands is likely to reflect the length of the repetitive GGAAT SatIII monomer. The lack of hybridization signals with the complementary probe (not shown), along with RNAse sensitivity and DNAse I resistance (Figure 2A), rule out the possibility that hybridization bands could correspond to DNA degradation products. Interestingly, the mechanism involved in the production of these molecules appears to be conserved in mammalian species, like hamster, that do not contain SatIII sequences in their genome. When pericentromeric SatIII DNA repeats are introduced into hamster cells (hamster-human somatic cell hybrid GM10611A bearing human chromosome 9—HSA9) heat shock-induced SatIII RNAs are expressed, while they are not detectable in parental hamster B14 cells [22]. As in HeLa cells, these molecules are relatively stable and remain above the basal level for at least 2 days (histogram in Figure 2B). Northern blotting analysis of small RNA preparations with the LNA probe detects a ladder of short SatIII RNAs, comparable to that observed in HeLa cells, in GM10611A but not in parental B14 cells (Figure 2B).

Altogether, these results are compatible with the idea that short SatIII RNAs could derive from the processing of pre-existing longer transcripts. To verify this hypothesis, we tested whether their level could be affected by Actinomycin D (ActD), a generic inhibitor of RNA polymerases. ActD (5 µg/mL) was added to heat-shocked HeLa cells at the border between phase II and III (22 h of recovery). Short RNAs were extracted and analyzed 8 h later. The level of long SatIII RNAs, as assessed using quantitative RT-PCR, is not perturbed by ActD as expected from the fact that they are synthesized during phase I of the heat shock response. In contrast, inhibition of transcription reduces the amount of the unstable *c-myc* control messenger (Figure 2C, lower panel). Notably, under these conditions, no difference is visible in the level of short SatIII RNAs between untreated and ActD treated cells (Figure 2C, upper panel), supporting our hypothesis that these molecules derive from the processing of longer pre-existing transcript rather than de novo transcription.

Since the long SatIII RNAs are exclusively nuclear, being associated with sites of transcription [36], we wondered whether also short SatIII RNAs could be nuclear. Therefore, we prepared short RNAs from chromatin enriched, nucleoplasm and cytoplasmic fractions purified from unstressed and heat shocked HeLa cells allowed to recover for 48 h at 37 °C. RNAs were then analyzed using Northern blotting with the SatIII LNA oligo. As a quality control of the purification procedure, RNAs were also probed with oligos against Mir16, mostly cytoplasmic, and U6 snRNA, which is present both in the cytoplasm and nucleoplasm. The results in Figure 2D indicate that most of the short SatIII RNAs are in the chromatin fraction, which also contains about 80% of long SatIII RNAs, as if processing of long into short molecules would occur in this fraction. Notably, no signal is visible in unstressed cells, even in the chromatin-enriched fraction, suggesting that under physiological conditions short SatIII RNAs are either absent or their level is below the threshold of detectability.

**Figure 3:**
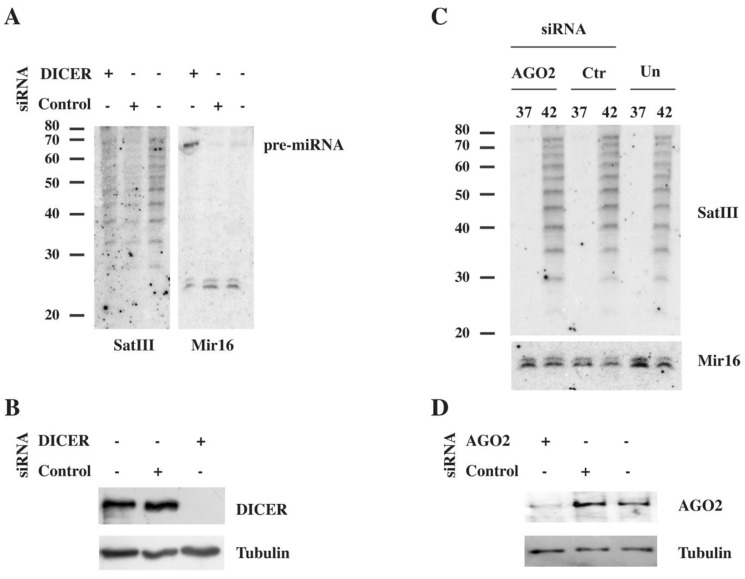
(**A**) HeLa cells untreated or transfected with DICER-specific or control siRNAs were heat shocked, and after 48 h, short RNAs were prepared and analyzed using Northern blotting, as in Figure 2. Mir16 was used as a loading control. The pre-miRNA level drastically increases after DICER down-regulation. (**B**) Total cell extracts from the same cells were analyzed using Western blotting to assess the efficacy of DICER down-regulation. Beta-tubulin was used as a loading control. (**C**) HeLa cells untreated (Un) or transfected with AGO2-specific or control (Ctr) siRNAs were heat shocked (42 °C), and after 48 h, short RNAs were prepared and analyzed using Northern blotting as in (A). Short RNAs from unstressed cells (37 °C) were also analyzed. Mir16 was used as a loading control. (**D**) Total cell extracts from the same cells were analyzed using Western blotting with anti-AGO2 and beta-tubulin antibodies.

Studies in different organisms suggest that noncoding RNAs deriving from transcription of centromeric and pericentromeric repetitive sequences are processed by DICER into short RNAs [37]. However, up to now there are no examples of a pattern comparable to the one described here. To understand whether DICER, even indirectly, could be involved in the production of short SatIII RNAs, we treated HeLa cells with a *DICER*-specific siRNA. After, two rounds of transfection cells were heat shocked for 1 h at 42 °C and allowed to recover for 48 h at 37 °C before harvesting. Down-regulation drastically decreases the abundance of DICER (Figure 3B) and affects the maturation of Mir16 (Figure 3A, right panel). However, no effect on the abundance and size distribution of short SatIII RNAs (Figure 3A, left panel) was detectable, arguing against the involvement of the RNA interference apparatus in the production of short SatIII RNAs. This conclusion was further supported by the observation that siRNA-mediated down regulation of Argonaute 2 (AGO2), which has a slicer activity and is able to cut target RNA molecules [38], does not affect the abundance and size distribution of short SatIII RNAs (Figure 3C,D).

### 2.3. Heat Shock Affects Cell Cycle Progression and the Execution of Mitosis

**Figure 4:**
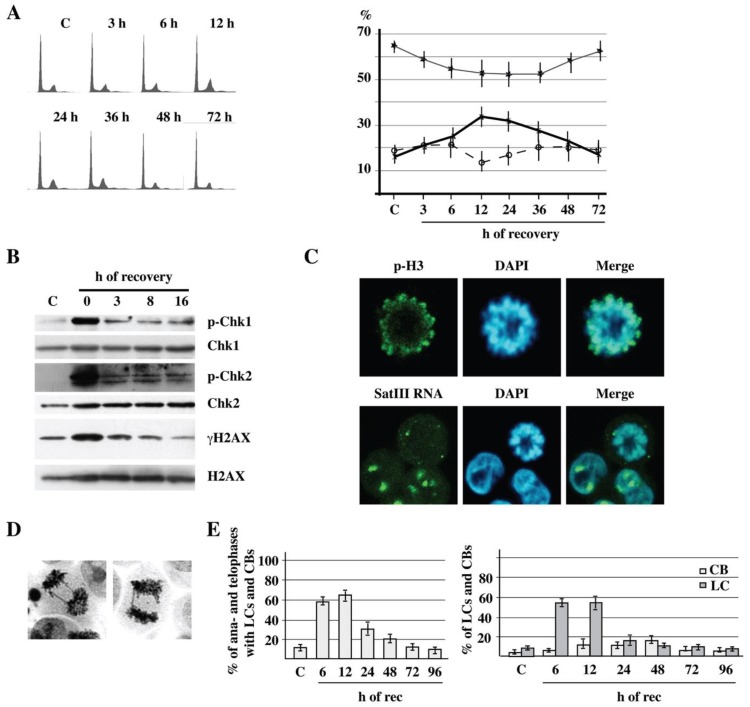
Heat shock affects cell cycle and mitotic progression. (**A**) FCM analysis of HeLa cells unstressed (C) or heat-shocked for 1 h at 42 °C and allowed to recover at 37 °C for the indicated time periods. The graph on the right shows the % of cells in G1 (black stars), S (white circles) and G2/M phases (X) in control (C) cells and at the indicated time points of recovery. Each value is the mean ± standard deviation of three independent experiments. (**B**) Western blot analysis of cell extracts prepared from control cells (C) and from cells allowed to recover for the indicated time periods. Extracts were analyzed with antibodies against Chk1 and Chk2 checkpoint kinases and the histone variant H2AX. The same filters were probed with antibodies against the phosphorylated forms of the same proteins (p-Chk1, p-Chk2 and γH2AX). (**C**) Heat-shocked HeLa cells were allowed to recover for 3 h at 37 °C and analyzed in immunofluorescence with an antibody against histone H3 phosphorylated on Ser10 or in RNA FISH with the reverse oligonucleotide probe specific for G-rich SatIII RNAs. Nuclei were counterstained with DAPI. Representative confocal laser images are shown. (**D**) Microscopy images of heat-shocked HeLa allowed to recover at 37 °C for 8 h and stained with haematoxylin. (**E**) Heat-shocked HeLa cells were allowed to recover at 37 °C for the indicated time periods, stained with DAPI and analyzed using confocal imaging. A total of 300 mitotic cells were counted in three independent experiments. The histogram shows the percentages of chromosomal bridges (CB) and lagging chromosomes (LC) ± standard deviation at different recovery times.

We wondered whether the three phases in the life of SatIII RNAs could be linked to other aspects of cell physiology potentially affected by thermal stress, as for instance cell cycle progression. Indeed, Flow cytometry analysis (Figure 4A) indicates that moderate heat shock (1 h at 42 °C) produces a progressive accumulation of HeLa cells in G2/M. The fraction of G2/M cells increases from 15% before stress to 34% at 12 h of recovery. Thereafter, cells progressively return to their normal distribution during phase III. This behavior clearly suggests a lasting problem connected with mitosis. Heat shock is known to induce replication-dependent DNA damage and the phosphorylation of the H2AX histone variant on serine 139 (γH2AX; [39]). In agreement with previous reports [39], phosphorylation of H2AX, Chk1 and Chk2 kinases increases immediately at the end of thermal stress (Figure 4B) and very rapidly reverts to basal level. Thus, thermal stress triggers the transient activation of genome surveillance mechanisms.

We decided to investigate the nature of the G2/M block using single-cell analysis. We noticed that cells reaching mitosis during the first part of phase I (up to 3 h of recovery) accumulate in prophase and pro-metaphase, which are marked by phosphorylation of histone H3 on serine 10 (Figure 4C). Interestingly, RNA FISH analysis unveils a complex distribution of SatIII RNAs in these cells. As exemplified in Figure 4C, a few SatIII foci remain associated with condensed chromosomes, while several small foci are detectable throughout the cell volume. This pattern is suggestive of a partial breakdown of nSBs, which may explain the reduction in number and size of these structures observed at 24 h of recovery (see Figure 1B).

This pro-metaphase block is transient and is no longer detectable at 6 h of recovery. However, serious mitotic problems are observed during the following two days. Indeed, cell staining with either hematoxylin (Figure 4D) or DAPI reveals the presence of aberrant mitoses with lagging chromosomes (LCs) and chromosomal bridges (CBs). A significant fraction of chromosome segregation defects is already visible in unstressed HeLa cells, with about 3% and 7% of anaphases/telophases showing CBs and LCs, respectively. However, more than 50% of anaphases/telophases show LCs at 6 h and 12 h of recovery. This figure rapidly declines to less than 20% at 24 h, and the fraction of mitoses with LCs is comparable to that observed in unstressed cells at the end of day 2. A different curve is observable in the case of CBs, since the proportion of anaphases/telophases with CBs peaks between day 1 and day 2 and never exceeds 20% (Figure 4E).

### 2.4. nSBs Are Often Associated with Lagging Chromosomes and Chromosome Bridges

**Figure 5:**
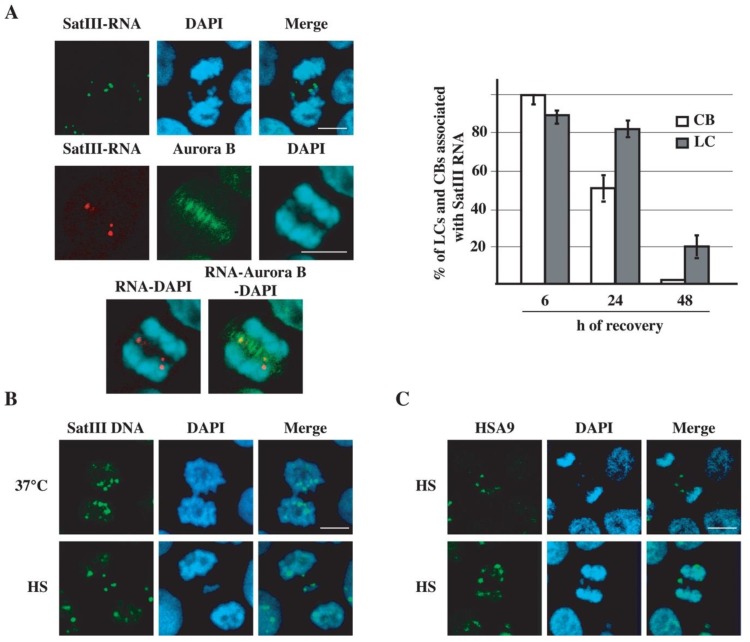
Heat shock affects segregation of human chromosomes bound to nSBs. (**A**) Heat-shocked HeLa cells were allowed to recover for 8 h and analyzed using RNA FISH with the reverse oligonucleotide probe specific for SatIII RNAs. Cells were co-stained with an antibody against Aurora B and counterstained with DAPI to identify cells in anaphase. Representative confocal laser images are shown. The histogram shows the percentage of CBs and LCs associated with SatIII RNAs at the indicated hours of recovery from stress. Percentages have been calculated in three independent experiments with a total of 100 mitoses. (**B**) Heat-shocked HeLa cells were analyzed using DNA FISH with an oligo probe specific for the C-rich strand of SatIII repeats or (**C**) with a whole chromosome painting probe for HSA9. Confocal laser images of HeLa cells allowed to recover for 8 h after heat shock. HS, heat shock. Nuclei were counterstained with DAPI. Bars: 10 µm.

The association of nSBs with chromosomes in prophase (Figure 4C) may explain how these nuclear bodies are inherited by daughter cells. Therefore, we decided to investigate the behavior of these bodies throughout mitosis, by staining the SatIII RNA with a biotinylated oligo. Surprisingly, during anaphase and telophase, in addition to being embedded in the two chromosomal sets, SatIII RNAs are also bound to LCs and CBs (Figure 5A). The histogram in Figure 5A indicates that at 6 h of recovery, 100% of CBs and 86% of LCs are marked by SatIII RNA foci. This fraction decreases with time, and at 48 h of recovery CBs are no longer bound, while 20% of LCs are still labelled by SatIII RNAs. However, the association with SatIII RNAs does not necessarily imply a defect in chromosome segregation, and a fraction of SatIII foci is embedded in the group of correctly segregating chromosomes.

In interphase, nSBs are assembled onto arrays of SatIII DNA repeats, such as those found in the extended pericentric heterochromatic q12 band of chromosome 9 [22]. To investigate whether LCs and CBs contained arrays of SatIII repeats, heat-shocked HeLa cells were analyzed in DNA FISH with a biotinylated oligo complementary to the C-rich strand of the SatIII sequence, which does not recognize SatIII RNAs and does not stain nSBs [23]. This analysis shows that 100% of LCs at 6 h and 24 h of recovery contain SatIII DNA arrays, while no labelling of LCs was visible in unstressed cells (Figure 5B). Concerning CBs, virtually all of them were stained by the oligo at 6 h of recovery, while SatIII sequences can be detected only in 43% of CBs at 24 h. Altogether, these data suggest a link between the presence of SatIII sequences and the chromosome segregation defects triggered by heat shock.

### 2.5. Mitotic nSBs Are Chromosome-Associated Ribonucleoprotein Complexes

**Figure 6:**
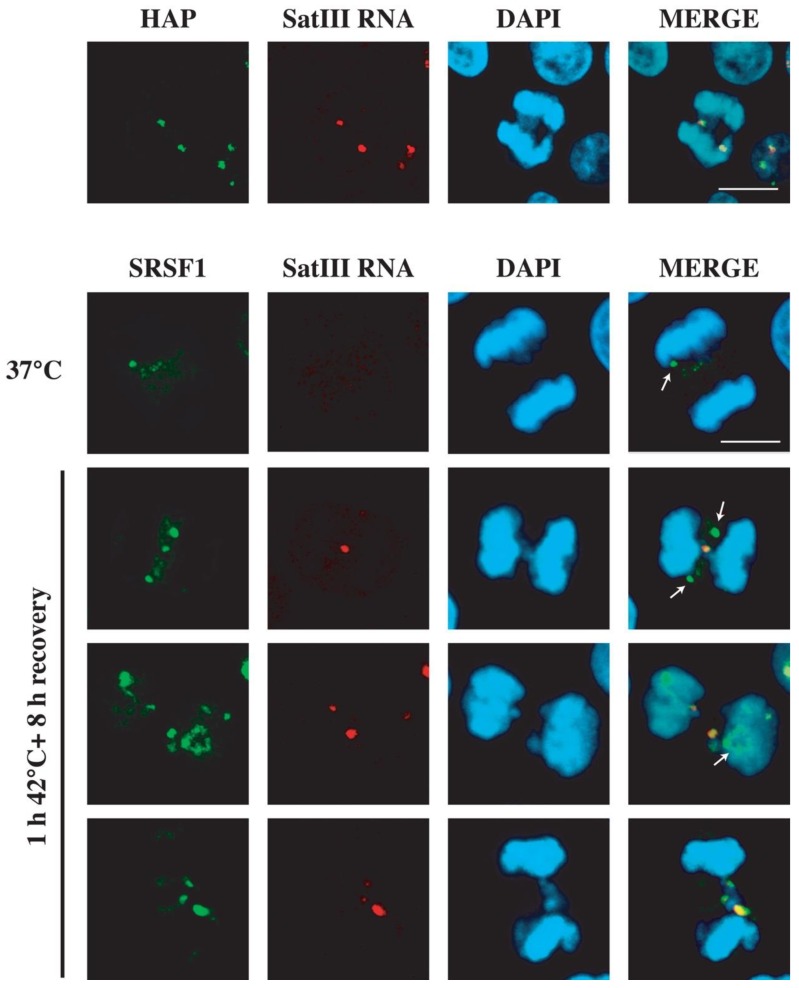
RNA binding proteins remain associated with SatIII RNAs on lagging chromosomes. Heat-shocked HeLa cells were allowed to recover for 6 h at 37 °C and then analyzed using RNA FISH with an oligo specific for SatIII RNAs and co-stained with antibodies specific for hnRNP HAP or splicing factor SRSF1. Nuclei were counterstained with DAPI. The arrows in lane “37 °C” and in the first lane “1 h 42 °C + 8 h recovery” point to mitotic interchromatin granules (MIGs). The arrow in the second lane “1 h 42 °C + 8 h recovery” points to nucleoli organizing region-associated patches (NAPs). Bars: 10 µm.

nSBs are enriched in proteins involved in pre-mRNA splicing that are recruited via SatIII RNAs interaction [40]. To verify whether RNA binding proteins are associated to SatIII RNAs bound to LCs and CBs, heat-shocked HeLa cells were allowed to recover for 8 h and stained with antibodies against two components of nSBs, namely hnRNP HAP/Saf-B and splicing factor SRSF1. As exemplified in Figure 6, both proteins colocalize with SatIII RNA in anaphase and telophase. Particularly interesting is the behavior of SRSF1, which in unstressed cells is a component of nuclear speckles. Nuclear speckles disassemble during mitosis and their constituents diffuse throughout the cytoplasm. During metaphase, splicing factors, including SRSF1, organize into still poorly defined structures called mitotic interchromatin granules (MIGs), which survive throughout anaphase (Figure 6, see arrows in 37 °C and in row of 8 h of recovery) and have been proposed to be equivalent of interphase nuclear speckles [41]. MIGs disappear at telophase when pre-mRNA processing factors accumulate in patches around nucleoli organizing regions (NOR) called NOR-associated patches or NAPs [42]. Interestingly, in cells recovering from thermal stress, a fraction of SRSF1 during telophase is still associated with nSBs bound to LCs, while most of the protein is already found in NAPs (Figure 6, see arrow in second row of 8 h of recovery).

**Figure 7:**
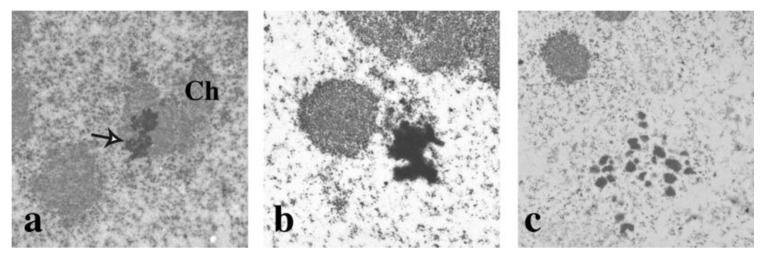
Electron microscopy analysis of mitotic cells harvested after heat shock. HeLa cells were heat shocked and allowed to recover at 37 °C for 3 h and then incubated for 18 h in the presence of nocodazole (80 ng/mL). Cells were analyzed using electron microscopy as previously described. Ch: chromatin. Arrow in panel (**a**) points to clusters of perichromatin granules embedded in chromatin. In panel (**b**) chromatin protrusions are visible linking the chromatin mass to clusters of perichromatin granules. Dispersed perichromatin granules are visible in panel (**c**).

To further investigate the relationship between nSBs in mitosis and in interphase nuclei, we studied their structure using electron microscopy (EM). Heat-shocked cells were allowed to recover for 3 h at 37 °C and then incubated for 15 h in the presence of nocodazole (80 ng/mL) to block the cells in pro-metaphase. EM images of nocodazole treated cells show the presence of large clusters of perichromatin granules (PG) similar to those described in interphase cells [28] and adjacent to or embedded in condensed chromosomes (Figure 7a,b). In some images (Figure 7b), a chromatin fiber appears to anchor the nSB to the chromosome, suggesting a distortion of the chromosome structure. Finally, groups of dispersed PGs, which probably represent disassembling bodies no longer associated with chromosomes, are visible in some fields (Figure 7c); these structures may correspond to the SatIII foci dispersed throughout the cell volume in Figure 3C. As expected, no PG clusters were visible in unstressed cells after nocodazole treatment.

## 3. Discussion

The function of SatIII RNAs and nSBs in the cell response to stress treatments, particularly heat shock, has long remained a matter of hypotheses. Only recently, a few studies shed new light on this phenomenon and proved that SatIII RNAs have a direct role in controlling gene expression during the initial phase of the recovery from stress by modulating gene transcription and alternative splicing programs [31,43].

The analysis of nSBs and SatIII RNAs reported here unveils unexpected facets of thermal stress that are not limited to a few hours following stress but last up to 4 days of recovery.

### 3.1. Heat Shock Affects Cell Biology Over a Long-Time Interval

The massive synthesis of SatIII RNAs and the consequent formation of nSBs identify the acute response to stress or phase I. The length of this period slightly differs among experiments, but it is certainly completed within the first 3–6 h of recovery from heat shock, which corresponds to the period commonly considered by studies in the field. Indeed, transcription factor HSF1 and RNAPII are not detectable in nSBs at longer recovery times [29,31]. Another distinguishing feature of phase I is the transient block of mitosis in prophase/prometaphase (Figure 4), which is then completely reverted. In the following 20 h (up to end of day 1, phase II), most SatIII RNAs are degraded, and only small nSBs are detectable in the majority of the cell nuclei (Figure 1). We have no clues about the molecular mechanism involved in the degradation of SatIII molecules. However, RNA FISH experiments (Figure 4C) indicate that a fraction of nSBs break down at mitosis and SatIII foci spread throughout the cell volume. A subset of nSBs, however, remains associated with mitotic chromosomes and is eventually inherited by daughter cells. Thus, it is plausible that passage through mitosis may play a role in the disassembly of nSBs and degradation of SatIII molecules. This is consistent with the observation that loose groups of perichromatin granules (i.e., the subunits of nSBs) [40] are detectable using electron microscopy in the interchromatin space of cells halted in prometaphase with nocodazole (Figure 7). Interestingly, chromosome-bound SatIII RNAs at the end of the first day of recovery are relatively stable and their level is still slightly higher than that in control cells by the end of day 4 (Figure 1). We hypothesize that a fraction of nSBs are lost at successive mitoses, thus contributing to progressive reduction in SatIII RNA molecules. However, our data suggest that an additional mechanism operates to lessen the level of chromatin-bound SatIII RNAs from day 2 to day 4. Indeed, during this long interval, which corresponds to the third and last phase of the heat shock response, long SatIII RNAs are processed into a ladder of discrete species of small RNAs ranging in size from 25 to less than 100 nt and showing a regular increment of 5 nt that most likely reflects the size of the GGAAT monomer of the SatIII repeat. Our data (Figure 2) suggest that processing affects the chromatin bound pool of transcripts, and in fact, short SatIII RNAs are actually associated with the chromatin-enriched fraction. Although the function of these short RNAs is still a matter of investigation, we hypothesize that they represent the final step in the disassembly of nSBs. Whether this phenomenon can be a model for RNA-based chromatin organization processes remains an attractive speculation. A strong body of evidence indicates that components of the RNAi machinery, first of all DICER and AGO2, play a main role in the production of short centromeric and pericentromeric RNAs in different species [37]. However, our data seem to rule out their role in the production of the ladder of short SatIII RNAs (Figure 3). The identification of the mechanism involved in this process could shed new light on the function of arrays of satellite III DNA.

### 3.2. Heat Shock Affects Chromosome Migration During Mitosis

In agreement with previous analyses [44,45], we have found that the M-phase is a main target of heat shock. Thermal stress induces DNA damage [46] and results in the transient activation of Chk1 [47] and Chk2 [48] checkpoint kinases. Notably, Chk1 activation delays mitotic progression in heat-shocked cells [45,49,50]. However, different setting conditions appear to block cells at different mitotic sub-phases. Thus, for instance, harsh heat shock (3 h at 45 °C) leads to prometaphase arrests via the formation of the multipolar spindle [45,49,50]. On the contrary, a milder stress (30 min at 42 °C) prolongs the duration of metaphase and prevents the onset of anaphase [51]. Under our experimental conditions (1 h at 42 °C), an intermediate effect is observed in which cells show a delay in prometaphase without any evidence of multipolarity. Consistently with previous analysis, we have found that thermal stress induces chromosome mis-segregation. It has been suggested that lagging chromosomes observed after very mild heat shock is linked to SAC (spindle assembly checkpoint) attenuation resulting from ATP shortage produced by heat stress [51].

As discussed ahead, our analysis suggests an alternative possibility which involves the transcriptional activation of SatIII sequences. We have observed that, while the prometaphase block is limited to the first 3 h of recovery, the execution of mitosis is perturbed for a long recovery period and this effect is accompanied by a profound alteration of the cell cycle profile. At 12 h from heat shock, the fraction of cells in G2/M is twice as large as that measured in unstressed HeLa cells and remains higher than in controls up to 48 h of recovery (Figure 4A). In the same interval a high frequency of mitotic errors is observed, as revealed by the appearance of CBs and LCs (see Figure 4), pointing to the existence of a link between thermal stress and chromosome instability (CIN), a common feature of cancer cells [33].

We have observed that heat shock induces a drastic increase in the fraction of mitoses with LCs and CBs (Figure 4). Moreover, the vast majority of LCs and CBs detectable during the recovery from moderate stress contain SatIII DNA and are decorated by SatIII RNAs in nSBs (Figure 5). No such preference is observed for LCs and CBs in unstressed HeLa cells. The association of SatIII RNAs with mitotic HSA9 has been already reported by the group of Claire Vourc’h [24]; however, these authors limited their analysis to phase I (3 h of recovery), which may explain why they did not detect mitotic errors. In addition to confirming previous analysis, our data suggest that heat shock affects mitotic segregation of chromosomes containing arrays of SatIII DNA, including HSA9. It has been recently shown that SatII sequences are also transcriptionally activated in response to heat shock, leading to the production of “secondary nSBs” [52]. Thus, any of the 14 human chromosomes containing pericentromeric arrays of SatII/SatIII repeats may possibly undergo migration defects upon heat shock. It is plausible that the massive activation of SatIII and SatII sequences has a direct role in this phenomenon.

It is now well-established that long noncoding RNAs (lncRNAs) transcribed from pericentric repeats play an important role in heterochromatin formation in mammalian cells [53] and aberrant levels of these RNAs result in genomic instability and cancer [17,18]. A few recent studies have started to uncover the molecular basis of this link [53,54]. Thus, transcription of α-satellite sequences embedded in pericentromeric regions generates chromatin-associated RNAs that promote the recruitment of SUV39H1 [53] in this manner, supporting the assembly of H3K9me3-dependent heterochromatin. Whether and how the massive transcription of SatIII sequences triggered by heat shock can hamper the assembly of heterochromatin mediated by nearby α-satellite sequences remains an interesting possibility that deserves further analysis. Interestingly, a drop in the level of pericentric H3K9me3 and heterochromatin organization promotes transcriptional activation of pericentromeric satellite sequences [55,56,57,58]. The assembly and maintenance of pericentromeric heterochromatin involves the action of different complexes. Thus, in addition to the α-satellite-SUV39H1-H3K9me3-HP1 pathway, SIRT6 operates in silencing of pericentric heterochromatin by controlling the H3K18 acetylation level and the recruitment of the transcriptional co-repressor KAPI [59]. Notably, SIRT6 down regulation is accompanied by accumulation of SatIII RNAs and by the occurrence of mitotic errors without affecting the level of pericentromeric H3K9me3. Interestingly, mitotic errors and lagging chromosomes occurring in SIRT6 depleted cells are rescued by the depletion of SatIII RNAs, proving a causative role of these transcripts in mitotic dysfunction and genome instability [59].

### 3.3. Heat Shock Targets the Distribution of RNA Binding Proteins in Mitosis

The behavior of RNA binding proteins and ribonucleoprotein complexes at mitosis is still poorly characterized. Upon breakage of the nuclear envelope, splicing factors diffuse throughout the cytoplasm [60]. In metaphase, components of the splicing machinery, including SR factors, gather in a few small structures known as mitotic interchromatin granules (MIGs), which are supposedly analogous to interchromatin granule clusters (IGCs) observed in interphase cells [41,61]. As mitosis progresses to anaphase, the MIGs increase in number and size and eventually, in late-telophase, after re-formation of the nuclear envelope, dissolve, and splicing factors enter daughter nuclei [61]. The function of these structures in mitotic cells is still unclear. However, the differential release of splicing factors from MIGs, with SRSF1 entering the cell nucleus in late telophase while SRSF2 in G1 [61], suggests a role of MIGs in modulating the modification of splicing factors before their nuclear entry (Prasanth). In this scenario, MIGs would function to prepare splicing factors for their immediate targeting to transcription sites [60]. From this viewpoint, our finding that the association of RNA binding proteins (SRSF1and hnRNP HAP/SAF-B) with SatIII RNA persists throughout mitosis may have functional implications. On one side, it indicates that ribonucleoprotein complexes bound to chromatin can survive mitosis in spite of all the molecular events promoting chromatin compaction. Moreover, it clearly indicates that the composition of MIGs can be targeted by heat shock with plausible consequences on alternative splicing programs in the ensuing G1 phase. This hypothesis is supported by the behavior of splicing factor SRSF1, which in anaphase appears to be distributed between MIGs and nSBs. Moreover, in telophase, in addition to the accumulation of patches around nucleoli organizing region (NOR) called NOR-associated patches or NAPs [42] as in control cells, SRSF1 remains associated to lagging chromosomes (Figure 6). Altogether, our observations unequivocally prove that the effects of heat shock on cell metabolism last for a long time period through consecutive cell divisions and open a new perspective on the cell response to stress.

## 4. Materials and Methods 

### 4.1. Cell Lines and Treatments

HeLa cells were grown in DMEM, 10% fetal bovine serum (Sigma), 50 µg/mL gentamicin and 2 mM L-glutamine (all cell reagents from Sigma-Aldrich, Milan, Italy). B14-150, Chinese hamster ovary cells and GM-10611A hamster>human somatic cell hybrids [22] were grown in RPMI-1640 (Sigma-Aldrich), 10% fetal bovine serum, 50 µg/mL gentamicin and 2 mM L-glutamine. Heat shock experiments were performed at 42 °C as previously described [62]. Actinomycin D (Sigma-Aldrich) was added to a final concentration of 5 µg/mL to block transcription. Metaphase enrichment for electron microscopy analysis was achieved by growing cells for 15 h in the presence of 80 ng/mL of nocodazole (Sigma-Aldrich) after heat shock. Flow cytometry was applied to assess cell distribution in different phases of the cell cycle. Measurements were performed using a Partec PAS II (Partec GmBH, Münster, Germany).

### 4.2. In Situ Hybridisation to RNA and DNA

RNA fluorescence in situ hybridization (RNA FISH) was performed as previously described [23,36]. Briefly, cells were fixed in 4% paraformaldehyde in PBS (Sigma-Aldrich) for 15 min and permeabilized in 0.5% Triton X-100 (Sigma-Aldrich) on ice for 5 min. Hybridisation was carried out overnight at 42 °C with 5 ng/mL 5′ biotinylated reverse oligonucleotide probe (5′-ATT CCA ATC CAT GCC ATT CC-3′) [23]. The biotinylated probe was detected with FITC-avidin (Vector Laboratories, Burlingame, CA, USA), and the signal was amplified with biotinylated anti-avidin D antibody (Vector Laboratories) followed by FITC-avidin. For DNA in situ hybridisation, cells were fixed in 3:1 methanol-acetic acid (Sigma-Aldrich) for 15 min, then slides were aged overnight in a dry stove at 37 °C. The following morning, cells were washed in 2× SSC (1× SSC: 0.15M NaCl, 0.015 M Na citrate pH 7.0) (Sigma-Aldrich) for 2 min and immediately treated with 100 µg/mL RNase A (Sigma-Aldrich) in 2× SSC at 37 °C for 1 h. Cells were rinsed in 2× SSC, dehydrated in an ethanol series (70%, 90% and 100%) for 5 min each at room temperature and then air-dried. Hybridization was carried out in 100 µL containing 6 ng/µL of oligonucleotide probe, 10% dextran sulphate (Sigma-Aldrich) 50% formamide, 2x SSC and 1% Tween-20 (Sigma-Aldrich). The biotinylated direct probe (5′-GGA ATG GCA TGG ATT GGA AT-3′) used in this assay was complementary to nucleotides 97–106 of the SatIII sequence (accession number: X06137). Probe and DNA were denatured simultaneously. The hybridisation was carried out overnight at 37 °C in a humid chamber. The biotinylated probe was detected with FITC-avidin (Vector Laboratories) and the signal was amplified with biotinylated antiavidin D antibody (Vector Laboratories) followed by FITC-avidin. Nuclei were stained with 0.1 µg/mL 4′-6-diamidino-2 phenylindole (DAPI, Sigma-Aldrich). For HAS9 painting, HeLa cells were grown on coverslips, heat shocked and allowed to recover for 15 h. Cells were fixed in 3:1 methanol-acetic acid for 10 min, and the slides were allowed to air dry. The probe mixture used was specific for whole chromosome 9 detection (Cytocell Ltd, Cambridge, UK). Hybridization was carried out at 37 °C overnight.

### 4.3. RNA Interference

RNA interference was performed as previously described [63]. Briefly, Hela cells were grown in Opti-MEM medium (Thermo Fisher Scientific, Waltham, MA, USA) with 10% FBS for 24 h. Then, 200 pmol of siRNA were transfected using 20 µL RNAiMAX Reverse Transfection Lipofectamine (Thermo Fisher Scientific). Transfection was performed using siGENOME ON-TARGETplus SMART pool siRNA (Horizon Discovery, Cambridge, UK), which consists of four nucleotide sequences targeted to the *DICER* or *AGO2* gene. A non-targeting pool of four different oligos (Horizon Discovery) was used as a control. Two cycles of siRNA transfection were performed with 24 h intervals. Six hours after the second transfection, cells were plated and the following day heat shocked.

### 4.4. Indirect Immunofluorescence

Indirect immunofluorescence was performed as previously described [28] on HeLa cells grown on coverslips and fixed for 20 min in 4% paraformaldehyde (Sigma-Aldrich) and permeabilized in 0.5% Triton X-100 for 7 min on ice. Primary antibodies used were affinity purified rabbit anti-HAP polyclonal antibody [62], rabbit anti-Aurora B polyclonal antibody (Abcam, Cambridge, UK), mouse mAb clone 103 anti SRSF1 (Thermo Fisher Scientific), rabbit anti phosphor-histone H3 (Mitosis Marker) (Thermo Fisher Scientific) and rabbit polyclonal to CENP-A (Cell Signaling, Leiden, The Netherlands). Secondary antibodies used were rhodamine-conjugated anti-rabbit, anti-mouse immunoglobulin (IgG) goat antibodies (Jackson ImmunoResearch Laboratories, Ely, UK) and fluorescein isothiocyanate (FITC)-conjugated anti rabbit or anti-mouse IgG goat antibodies (Jackson ImmunoResearch). Nuclei were counterstained with DAPI, rinsed and mounted with fluorescent mounting medium (DakoCytomation California, Carpinteria, CA). Confocal microscopy was performed with a TCS-NT digital scanning confocal microscope (Leica, Deerfield, IL) equipped with a 63 X/NA = 1.32 oil immersion objective. We used the 488 nm laser line for excitation of FITC (detected at 500 nm < FITC < 540 nm) and the 543 nm laser line for the rhodamine fluorescence (detected at >590 nm). Images were exported to Adobe Photoshop (Adobe System, Mountain View, CA). For conventional microscopy, samples were analyzed with an Olympus IX71 microscope (Segrate, Italy), and images were acquired with a Roper scientific Photometrix digital camera (Tucson, AZ, USA) using the Metamorph 6.2r2 software (Molecular Devices San Jose, CA, USA) and exported to Adobe Photoshop (Adobe System, Mountain View, CA, USA).

### 4.5. Hematoxylin Staining

For hematoxylin staining, heat-shocked cells were washed once with PBS and fixed in 3:1 methanol-acetic acid for 10 min. Slides were air dried and aged overnight in an oven at 37 °C. Cells were stained with haematoxylin solution (Sigma-Aldrich) for 8 min, washed in distillate water and air dried.

### 4.6. Total RNA Extraction, RT-PCR and Quantitative RT-PCR (qRT-PCR)

Total RNA was purified from HeLa cells using an RNeasy Kit (Qiagen, Dusseldorf, Germany), following the manufacturer’s protocol. Subsequently, the RNA was treated with Turbo DNase (Thermo Fisher Scientific) for 30 min at 37 °C, to remove any contaminating DNA. RNA (1 µg) was reverse transcribed with MuLV reverse transcriptase (Thermo Fisher Scientific) with oligo (dT) or sequence-specific primers [25]. An aliquot (1/10th) of the reaction was then used in a quantitative PCR with QuantiTect SYBR Green PCR kit (Qiagen). PCR was carried out on Roche Light Cycler 480 (Roche, Monza, Italy). For RT-PCR analysis of *c-myc* and ribosomal *hPO* we used the following primers: hPO-F 5′-ATGCCCAGGGAAGACAGGGCG-3′; hPO-R 5′- CGAAGGGACATGCGGATCTGCTGC-3′; c-myc-F 5′-TGAGGAGACACCGCCCAC-3′; c-myc-R 5′- CAACATCGATTTCTTCCTCATCTTC- 3′.

### 4.7. Small RNAs Extraction and Northern Blot Analysis

Small RNAs were isolated using a mirVana^TM^ miRNA Isolation Kit (Thermo Fisher Scientific). To get rid of DNA, RNA samples were treated with Turbo DNase (Thermo Fisher Scientific). Northern blot protocol was inspired by the publication of [64] and then modified as indicated below. Small RNAs were separated on a denaturing 12% polyacrylamide gel, with 2 µg of RNA loaded per lane. Gel run was carried out at 2500 V and 35 W for 30 min in 0.5 X TBE. After gel running, samples were electrotransferred to a Zeta-Probe GT membrane (BioRad, Segrate, Italy). The transfer buffer was 0.5 X TBE. The blotting was carried out at 20 V and 0.36 A for 30 min. RNA molecules were then cross-linked to the membrane using UV irradiation at 1200 µj/cm^2^ (UVP CL1000 Longwave crosslinker Thermo Fisher Scientific). Pre-hybridization and hybridization solutions were used with LNA probes: 5× SSC, 50% formamide, 0.5% SDS, 5× Denhardt’s solution, 125 µg/mL tRNA. After overnight hybridization, membranes were washed several times up to the maximal stringency obtained with 0.1× SSC, 0.1% SDS for 5 min at 45 °C. Pre-hybridization and hybridization solution for oligonucleotide probes: 6 X SSC, 0.1% SDS, 10 X Denhardt’s solution, 125 µg/mL tRNA, 25 mM Na-phosphate. The most stringent wash was in 3 X SSC, 0.1% SDS for 30 min at room temperature. Oligonucleotide probes used were miR16 (5′-CGC CAA TAT TTA CGT GCT GCT-3′), U6 snRNA (5′-GAA TTT GCG TGT CAT CCT TGC GCA GGG GCC ATG CTA A-3′). Custom miRCURY^TM^ LNA Probes (Qiagen): Sat III C-rich (5′-ATT CCA TTC CAT TCC ATT CC-3′), Sat III G-rich (5′-GGA ATG GAA TGG AAT GGA AT-3′), molecular size marker: Decade^TM^ Marker System (Thermo Fisher Scientific). Oligonucleotides were labelled with α-^32^P ATP (3000 Ci/mmol) (Perkin Elmer, Waltham, MA, USA) and T4 polynucleotide kinase (Thermo Fisher Scientific) as previously described [65]. Membranes were scanned with a TYPHOON phosphorimager (GE, Marlborough, MA, USA) and images exported to Photoshop.

### 4.8. Cell Fractionation

HeLa cells were scraped off the flask and centrifuged at 4 °C for 5 min at 600× *g*. Cells were lysed in 10 volume of cold lysis buffer (0.15 M NaCl, 1.5 mM MgCl_2_, 10 mM Tris-HCl, pH 8.0, 0.4 U/μL of RNase inhibitor and 0.5% Nonidet P-40) (all chemicals are from Sigma-Aldrich). After 10 min on ice, cells were vortexed for 15 s and centrifuged at 4 °C for 3 min at 1000× *g* to separate the cytoplasm (supernatant) from nuclei (pellet). Nuclei were resuspended in 10 volume of lysis buffer and disrupted using mild sonication. The nucleoplasm (supernatant) was separated from the chromatin fraction (pellet), by spinning in Eppendorf centrifuge (Eppendorf, Milano, Italy) three times at 6000 rpm for 2 s. RNA was purified from fractions using an RNeasy Qiagen kit (Qiagen).

### 4.9. Western Blotting Analysis

Cells were lysed in Laemmli buffer supplemented with protease inhibitors (complete tablet; Roche) and phosphatase inhibitors (PhosSTOP tablet; Roche) and analyzed using Western blotting with the following primary antibodies: anti-DICER (Abcam); anti-AGO2 (Abcam); anti-α-Tubulin (Abcam), mouse monoclonal to phosphor-Histone H2AX (Ser 139) clone JBW301 (Thermo Fisher Scientific), rabbit polyclonal to Histone H2A (Thermo Fisher Scientific), rabbit polyclonal to phosphor-Chk2 (Thr68) (Cell Signaling, Denvers, MA, USA), mouse monoclonal to Chk2, clone 7 (Thermo Fisher Scientific), rabbit polyclonal to phosphor-Chk1 (Ser 354) (Cell Signaling) and mouse monoclonal to Chk1 (Santa Cruz, Heidelberg, Germany). Primary antibodies were revealed with peroxidase-conjugated goat anti-mouse (Jackson ImmunoResearch Laboratories) and an enhanced chemiluminescence system (Super Signal West Pico Pierce or Super Signal West Dura Extended; Thermo Fisher Scientific).

### 4.10. Electron Microscopy Analysis

Heat-shocked HeLa cells were harvested using trypsinization, immediately fixed in 4% formaldehyde (2 h at 4 °C) in the culture medium and then incubated in 2% OsO4 (Sigma-Aldrich) for 1 h at room temperature. Cell pellets were embedded in agar (Sigma-Aldrich) (2% in H_2_O), rinsed several times with S-rensen buffer (pH 7.2) (Sigma-Aldrich), and dehydrated in ethanol. Finally, the cells were embedded in LR White resin (Sigma-Aldrich) and polymerized at 60 °C for 24 h. Thin sections from formaldehyde-fixed cells were collected on nickel grids covered with a Formvar-carbon film (Sigma-Aldrich) and stained using the EDTA (Ethylenediaminetetraacetic acid) technique [66]. Specimens were observed with an EM900 electron microscope (Zeiss, Jena, Germany) equipped with a 30 µm objective aperture and operating at 80 kV.

### 4.11. Statistical Analysis

Data in all experiments were represented as mean ± standard deviation (SD) of three independent experiments.
